# Probing the Interaction of the Diarylquinoline TMC207 with Its Target Mycobacterial ATP Synthase

**DOI:** 10.1371/journal.pone.0023575

**Published:** 2011-08-17

**Authors:** Anna C. Haagsma, Ioana Podasca, Anil Koul, Koen Andries, Jerome Guillemont, Holger Lill, Dirk Bald

**Affiliations:** 1 Department of Molecular Cell Biology, Faculty of Earth and Life Sciences, VU University Amsterdam, Amsterdam, The Netherlands; 2 Amsterdam Institute for Molecules, Medicines and Systems (AIMMS), Amsterdam, The Netherlands; 3 Department of Antimicrobial Research, Tibotec NV, Johnson & Johnson Pharmaceutical Research and Development, Beerse, Belgium; 4 Department of Medicinal Chemistry, Janssen Research & Development, Johnson & Johnson, Val de Reuil, France; University of Cambridge, United Kingdom

## Abstract

Infections with *Mycobacterium tuberculosis* are substantially increasing on a worldwide scale and new antibiotics are urgently needed to combat concomitantly emerging drug-resistant mycobacterial strains. The diarylquinoline TMC207 is a highly promising drug candidate for treatment of tuberculosis. This compound kills *M. tuberculosis* by binding to a new target, mycobacterial ATP synthase. In this study we used biochemical assays and binding studies to characterize the interaction between TMC207 and ATP synthase. We show that TMC207 acts independent of the proton motive force and does not compete with protons for a common binding site. The drug is active on mycobacterial ATP synthesis at neutral and acidic pH with no significant change in affinity between pH 5.25 and pH 7.5, indicating that the protonated form of TMC207 is the active drug entity. The interaction of TMC207 with ATP synthase can be explained by a one-site binding mechanism, the drug molecule thus binds to a defined binding site on ATP synthase. TMC207 affinity for its target decreases with increasing ionic strength, suggesting that electrostatic forces play a significant role in drug binding. Our results are consistent with previous docking studies and provide experimental support for a predicted function of TMC207 in mimicking key residues in the proton transfer chain and blocking rotary movement of subunit c during catalysis. Furthermore, the high affinity of TMC207 at low proton motive force and low pH values may in part explain the exceptional ability of this compound to efficiently kill mycobacteria in different microenvironments.

## Introduction

Tuberculosis causes approximately 2 million deaths per year and an estimated 1/3 of the world population harbors *Mycobacterium tuberculosis* in a dormant or latent form [1], [2]. Infections with multidrug-resistant and extensively drug-resistant mycobacterial strains as well as co-infection with HIV pose a global health challenge [3]–[5]. Existing drug regimens need to be administered for at least 6 month, and up to 24 months in case of drug-resistant tuberculosis [4], [6]. To counteract development of drug-resistant strains and to shorten tuberculosis treatment the discovery of new drugs, validation of new target proteins, and understanding of drug/target interactions are essential [6]–[8].

**Figure 1:**
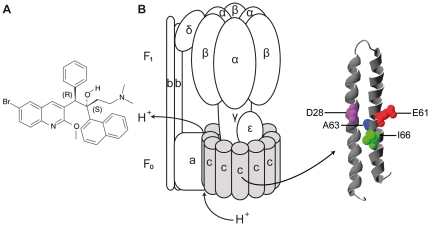
TMC207 and its target mycobacterial ATP synthase. (**A**) Structure formula of TMC207. (**B**) ATP synthase subunit composition with subunit c in grey. A homology model of a subunit c monomer from *Mycobacterium tuberculosis* is shown enlarged. The acidic residue Glu61, essential for proton transport, is depicted in red. Point mutations that influence mycobacterial sensitivity for TMC207 are indicated in colour.

Energy metabolism has emerged as a new target-pathway for development of new anti-tubercular drugs [8], [9]. The diarylquinoline TMC207 (Figure 1A) is a highly promising candidate for treatment of drug-resistant tuberculosis and for shortening of tuberculosis treatment [10]–[12]. TMC207 acts on a novel target, mycobacterial ATP synthase [13] and is highly active on replicating as well as on dormant mycobacteria [14], [15]. In phase II clinical trials addition of TMC207 to standard therapy antibacterial regimens strongly accelerated conversion to a negative sputum culture as compared to placebo [16]. TMC207 acts in a highly selective manner, with only minimal effect on human ATP synthase and only minor side effects in human patients [10], [16], [17].

ATP synthase is a ubiquitous key enzyme in energy metabolism of virtually all cells that utilizes the energy stored in a trans-membrane electrochemical potential difference of a coupling ion for production of ATP [18]. In mycobacteria, ATP synthase has been proven essential for growth on both fermentable as well as non-fermentable carbon sources [19]. Bacterial ATP synthase is composed of a membrane-embedded F_0_ sector with the subunit composition a_1_b_2_c_10–15_ and a hydrophilic F_1_ part, consisting of subunits a_3_β_3_γδε (Figure 1B). Proton flow through F_0_ triggers rotation of the oligomeric subunit c ring that is coupled to rotation of the γ subunit within the (αβ)_3_ hexamer of F_1_ and finally drives synthesis of ATP [20]–[22]. A significant step in proton transport is proton binding to an essential acidic residue in the central, trans-membrane part of subunit c [23]. TMC207 binds to purified mycobacterial subunit c [13] and mycobacterial sensitivity for TMC207 is influenced by point mutations located in the vicinity of the acidic residue in subunit c (Glu61 in *M. tuberculosis*, Figure 1B) [10], [13], [24]–[26]. These findings suggest that TMC207 may bind in that central, mostly hydrophobic part of subunit c. Based on docking studies it has been proposed that TMC207 binds at the interface of subunits c and subunit a [27]. The drug is predicted to mimic a conserved basic residue in the proton transfer chain, arginine186, subsequently interfering with the rotary movement of subunit c. [27]. However, no high-resolution structure is available for mycobacterial ATP synthase or its subunits. Moreover, biochemical data on TMC207/target interaction to test the predictions from the docking studies are scarce.

In the present report we used biochemical assays and binding studies to investigate the mode of binding between TMC207 and mycobacterial ATP synthase. We study factors potentially influencing drug/target interaction, such as the proton motive force, the pH value and buffer ionic strength. The results are correlated with proposed models for the TMC207 binding site and discussed in view of TMC207 being active in different microenvironments.

## Materials and Methods

### Bacterial strains and growth conditions

*Mycobacterium smegmatis* mc^2^155 was kindly provided by B.J. Appelmelk, Department of Molecular Cell Biology & Immunology, VU University Medical Center Amsterdam, the Netherlands. Replicating cultures of *M. smegmatis* were grown in Middlebrook 7H9 broth (Difco) with 10% Middlebrook albumin dextrose catalase enrichment (BBL) and 0.05% Tween-80 at 37°C to the late exponential phase.

### Preparation of inverted membrane vesicles

Inverted membrane vesicles (IMVs) of *M. smegmatis* were prepared as described previously [13]. Briefly, cells were pelleted by centrifugation at 5000 *g* for 20 min and washed once with Phosphate-buffered saline (PBS, pH 7.4). Five grams of cells (wet weight) were resuspended in 10 ml of 50 mM MOPS-KOH (pH 7.5), 2 mM MgCl_2_ including protease inhibitors (complete, EDTA free; protease inhibitor cocktail tablets from Roche). Lysozyme (1.2 mg/ml), 1500 units of deoxyribonuclease I (Invitrogen) and 13 mM MgCl_2_ were added and cells were incubated with stirring at room temperature for 45 minutes. The cells were broken by three passages through a pre-cooled French pressure cell at 20000 psi (Thermo Electron, 40K). The lysate was centrifuged at 5000 *g* and 4°C for 20 min to remove unbroken cells. The supernatant was centrifuged at 370000 *g* and 4°C for 1 h and the pellet of IMVs was washed with 50 mM MOPS-KOH (pH 7.5), 2 mM MgCl_2_. After the second centrifugation step, the inverted membrane fraction was resuspended in an appropriate volume of 50 mM MOPS-KOH (pH 7.5), 2 mM MgCl_2_.

### Assay of ATP synthesis

ATP synthesis activity was measured as described previously [17] with the modifications described below. IMVs (1 mg/ml) were incubated in either 50 mM MES-KOH (pH 5.25) or 50 mM MOPS-KOH (pH 6.0–7.5) containing 2 mM MgCl_2_, 2 mM ADP, 20 mM KH_2_PO_4_, 100 µM P^1^,P^5^-di(adenosine-5′) pentaphosphate (Ap5A), 25.4 mM glucose, 11.8 U/ml hexokinase (Sigma) and protease inhibitors (complete, EDTA-free; protease inhibitor cocktail tablets from Roche). To manipulate the proton motive force samples were supplemented with varying concentrations of uncoupler SF6847. Samples (0.25 ml) were incubated with vigorous stirring in 18-ml flasks at 37°C. The concentration of NADH to initiate the reaction was varied between 5–15 mM. After 1 h, each reaction was stopped with 25 mM EDTA, followed by transfer to ice. Samples were transferred to Eppendorf tubes, boiled for 5 min and centrifuged (10000 *g*, 20 min) to remove denatured protein. In the supernatants, the synthesized glucose-6-phosphate was oxidized by 2.5 mM NADP in the presence of 3 U/ml of glucose-6-phosphate dehydrogenase (Roche). NADPH formation was monitored using a spectrophotometer at 340 nm. The 50% inhibitory concentrations (IC_50s_) were determined using GraphPad Prism version 5.00 for Macintosh, GraphPad Software, San Diego California USA. The data were fitted with a model describing a one-site binding hyperbola.

### BIAcore binding studies

Binding studies using Surface Plasmon Resonance technology were carried out using a BIAcore 2000 machine with a carboxymethyl (CM-5) analytical chip. An amine-analog of TMC207, which carries an amino group instead of the bromine [13], was bound to the chip at 25°C as follows. 30 µl of an equimolar mixture of 1-ethyl-3-(3-dimethylaminopropyl) carbodiimide hydrochloride (EDC) and N-hydroxysuccinimide (NHS) was used to activate the carboxy-methyl surface of the chip. Subsequently, 30 µl of the TMC207 amine analog (50 µM) in 10 mM Hepes-KOH (pH 7.5), 2 mM MgCl_2_, 150 mM NaCl was bound to the activated chip at a flow rate of 2 µl/min. Non-reacted activated EDC/NHS on the chip surface was blocked by the infusion of 50 µl of 1 M ethanolamine.

Subunit c from *Mycobacterium tuberculosis* was purified as described in previously [13]. The purified subunit c (13 µM) was injected onto the compound-linked Biacore chip at a flow rate of 30 µl/min in 10 mM Hepes-KOH (pH 7.5), 2 mM MgCl_2_, 0.5% Triton X-100 containing 50 mM, 150 mM or 300 mM NaCl (37°C). Association, dissociation and equilibrium dissociation constants were determined using GraphPad Prism version 5.00 for Macintosh, GraphPad Software, San Diego California USA.

## Results and Discussion

### TMC207 does not compete with protons for a common binding site

We investigated the effect of the proton motive force on ATP synthesis inhibition by TMC207. TMC207 may interfere with ATP synthesis by competing with protons for the same binding site on ATP synthase. A high proton motive force may then outcompete TMC207 from its binding site, leading to reduced drug/target binding. Conversely, a decreased proton motive force then would lead to increased TMC207 binding. Moreover, the proton motive force not only supplies the energy required for synthesis of ATP, but also constitutes an important factor regulating the conformation of ATP synthase (for review see [28]). Consequently, the affinity of several known ATP synthase inhibitors depends significantly on the proton motive force [29]–[31].

**Figure 2:**
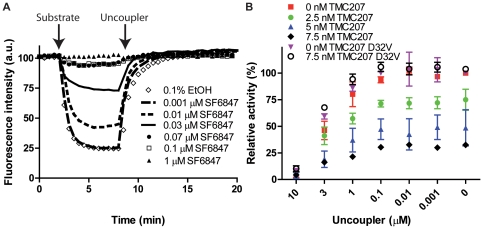
ATP synthesis inhibition by TMC207 at low proton motive force. (**A**) Inverted membrane vesicles from *Mycobacterium smegmatis* were diluted to 0.18 mg/ml in buffer containing 2 µM ACMA. To detect the proton motive force, quenching of ACMA fluorescence was investigated after addition of 5 mM succinate in the presence of increasing concentrations of the uncoupler SF6847. At the indicated time point, 1 µM of uncoupler SF6847 was added as control to collapse the proton gradient. (**B**) ATP synthesis by membrane vesicles of *M. smegmatis* (1 mg/ml) was measured in the presence of TMC207 and varying concentrations of uncoupler SF6847 to modulate the proton motive force. Samples were incubated at 37°C for 1 h in the presence of an ADP-regenerating system, and produced ATP was quantified spectrophotometrically by monitoring oxidation of glucose-6-phosphate with NADP^+^. As a control, 100 µM DCCD was added.

The proton motive force across inverted membrane vesicles (IMVs) of *Mycobacterium smegmatis* was monitored with the ACMA quenching method as in [32] and modulated using an uncoupler, SF6847 (Figure 2A). As expected, with increasing uncoupler concentration the ATP synthesis activity decreased in a dose-dependent manner, with <10% residual activity in the presence of 10 µM uncoupler (Figure 2B). We then tested three selected concentrations of TMC207 (2.5 nM, 5 nM and 7.5 nM), which in the absence of uncoupler decreased ATP synthesis activity by respectively 25%, 50% and 67%. As depicted in Figure 1B the inhibitory effect of TMC207 did not significantly change at lower proton motive force. The drug concentrations for half-maximal inhibition (IC_50_) values were determined to 5.0–7.5 nM TMC207 for all three uncoupler concentrations investigated. As a control, for membrane vesicles carrying the resistance mutation D32V in subunit c [10], no inhibition by 7.5 nM TMC207 was detected (Figure 2B). As a further control, inhibition by sodium azide (10 mM), an inhibitor known to act in a proton motive force dependent manner [29], [30], increased from <5% in the absence of uncoupler to >50% in the presence of the highest uncoupler concentration (data not shown). Thus, the proton motive force does not significantly influence the target's ability for binding of TMC207, neither by inducing conformational changes in ATP synthase, nor by outcompeting TMC207 from its binding site. These data strongly suggest that TMC207 does not directly compete with protons for a common binding site.

### TMC207 inhibits ATP synthesis at low and neutral pH values

**Figure 3:**
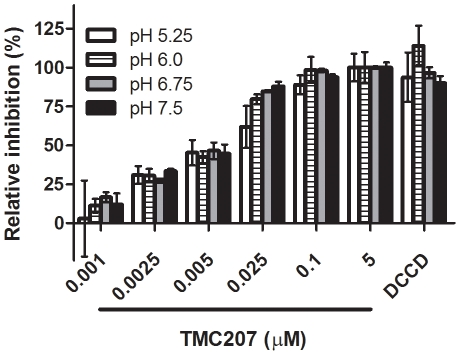
Effect of TMC207 on mycobacterial ATP synthesis at low pH. ATP synthesis in the presence of TMC207 and varying external pH values was measured for *Mycobacterium smegmatis* inverted membrane vesicles (1 mg/ml). Samples were incubated at 37°C for 1 h in the presence of an ADP-regenerating system, and produced ATP was quantified spectrophotometrically by monitoring oxidation of glucose-6-phosphate with NADP^+^. As a control, 100 µM DCCD was added.

Next, we investigated if (de-) protonation of TMC207 or of mycobacterial ATP synthase affects drug/target interaction. The membrane vesicles from *M. smegmatis* were capable of detectable ATP synthesis activity over the whole pH range investigated (pH 5.25–pH 7.5). As shown in Figure 3, the external pH did not significantly influence the inhibitory action of TMC207 between pH 5.25–7.5, with IC_50_ values determined to 5.0–7.5 nM for all pH values tested. Thus, neither (de-) protonation of TMC207 nor (de-) protonation of the target in the pH range investigated significantly changed the drug's ability to interact with its target. The dimethyl-amino group of TMC207 can take up a proton, which can be observed by a peak shift from 1.87 ppm to 2.18 ppm in the ^1^H NMR spectrum (data not shown). A pK_a_ value of 9.0–10.0 in aqueous solution is predicted, although this value may be lower in a hydrophobic membrane environment. Most of the inhibitor molecules will be protonated at neutral or acidic pH and the concentration of protonated TMC207 will not change significantly during a titration between pH 5.25 and 7.5 (<1.1fold increase according to the Henderson-Hasselbalch equation, assuming a pK_a_ of 9.5). The concentration of unprotonated TMC207 is expected to decrease strongly from pH 7.5 to pH 5.25 (>100fold according to the Henderson-Hasselbalch equation). The lack of pH dependency observed in our experiments thus suggests that the protonated form of TMC207 is the active drug entity.

### Electrostatic interactions are important for binding of TMC207

Docking studies predict that electrostatic interactions play an important role in binding of TMC207 to ATP synthase [27], [33].

**Figure 4:**
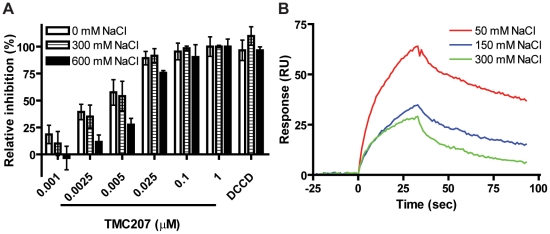
Electrostatic interactions are important for binding of TMC207. (**A**) ATP synthesis in the presence of TMC207 and increasing sodium chloride concentrations was measured for inverted membrane vesicles of *Mycobacterium smegmatis* (1 mg/ml). Samples were incubated at 37°C for 1 h in the presence of an ADP-regenerating system, and produced ATP was quantified spectrophotometrically by monitoring oxidation of glucose-6-phosphate with NADP^+^. As a control, 100 µM DCCD was added. (**B**) BIAcore binding studies. Purified subunit c from wild-type *Mycobacterium tuberculosis* was injected onto a chip with immobilized amine analog of TMC207 in the presence of 50, 150, and 300 mM NaCl at 37°C.

To test this prediction, we determined the effect of buffer ionic strength on TMC207 action. ATP synthesis by *M. smegmatis* membrane vesicles was susceptible to TMC207 at all ionic strengths conditions investigated (0 mM, 300 mM and 600 mM NaCl) (Figure 4A). However, sensitivity was clearly lower at high ionic strength, with IC_50_ values increasing from 3.9 nM at 50 mM NaCl, 5.0 nM (300 mM NaCl) to 12.9 nM (600 mM NaCl).

To support this finding, we used Surface Plasmon Resonance Sensing to characterize the interaction between TMC207 and purified ATP synthase subunit c. For these experiments we used an analog of TMC207, which carries an amino group instead of a bromine group [13] and thus can be conveniently linked to a BIAcore chip. As shown in Figure 4B, subunit c from *M. tuberculosis* bound to this TMC207 amino-analog [13] linked onto the chip. Subsequently, we tested the influence of various the salt concentrations (50–300 mM) in the subunit c sample and in the running buffer on drug/target interaction. Increasing concentrations of shielding ions significantly decreased binding affinity (Figure 4B). The equilibrium dissociation constants (*K_D_*) in the presence of 50 mM, 150 mM and 300 mM NaCl were determined to 1.5 µM, 4.2 µM and 19.7 µM, respectively. The deviation between K_D_ values and IC_50_ values may be explained by subunit a contributing to the TMC207 binding site as well [13], [27], [33]. The stronger ionic strength dependency observed in the BIAcore binding assays may be due to full accessibility of both drug and target for the salt ions, whereas in the membrane vesicle ATP synthesis assay the binding site of TMC207 supposedly is less accessible for the salt ions.

Our results suggest that electrostatic forces are an important factor for binding of TMC207 to ATP synthase, more specifically to its subunit c.

### TMC207 binds to a distinct binding site in ATP synthase

**Figure 5:**
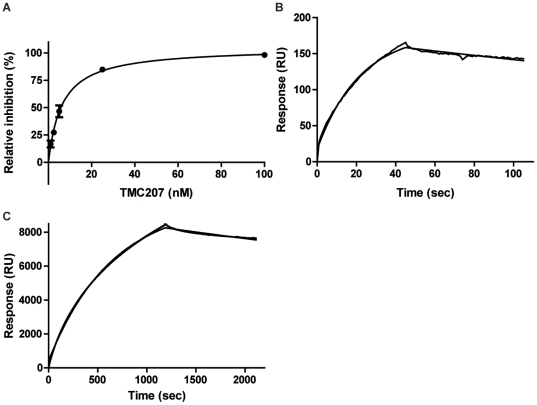
TMC207 binds to a defined binding site in ATP synthase. (**A**) The dose-dependency of ATP synthesis inhibition by TMC207 in inverted membrane vesicles of *Mycobacterium smegmatis* was fitted with a one-site binding hyperbola (Y = 104.9X/6.3+X, R^2^>0.99) (**B**) Binding of purified ATP synthase subunit c from *Mycobacterium tuberculosis* to an amine analog of TMC207 linked onto a BIAcore chip was fitted using mono-exponential binding models (Association = Req*(1−exp(−1*53737X)) and Dissociation = 165.654*exp(−1*0.002295*(X−45)) R^2^>0.99) and (**C**) Binding of purified ATP synthase from *Bacillus* PS3 to an amine analog of TMC207 linked onto a BIAcore chip was fitted using mono-exponential binding models (Association = Req*(1−exp(−1*153.7X)) and Dissociation = 8575.97*exp(−1*0.0001030*(X−1187)) R^2^>0.99).

ATP synthase is a complex membrane protein and in particular its membrane spanning regions may provide multiple binding sites for a predominantly hydrophobic molecule, such as TMC207. Therefore, we investigated if the inhibition of synthesis by TMC207 can be explained by binding of a single molecule TMC207 per ATP synthase complex. As depicted in Figure 5A, the dose-dependent inhibition of the ATP synthesis by TMC207 could be fitted accurately (R^2^>0.99) with a simple one-site saturation-binding curve. This indicates that interaction of TMC207 with a distinct binding site in ATP synthase is responsible for inhibition of ATP synthesis.

In order to corroborate this result, we also determined if binding of TMC207 to purified subunit c from *M. tuberculosis* or to the purified ATP synthase holoenzyme from *Bacillus* PS3 is consistent with a one-site binding mechanism. The Surface Plasmon Resonance studies showed that both *M. tuberculosis* subunit c and *Bacillus* PS3 ATP synthase bound to the TMC207 amine-analog immobilized on a BIAcore chip (Figure 5B and C). In both cases the obtained binding curves could be fitted well (R^2^>0.99) with a simple mono-exponential model, indicating only one type of binding site (Figure 5B and C). Taken together, our results suggest that TMC207 binds to a distinct drug-binding site in mycobacterial ATP synthase, most likely one molecule TMC207 is sufficient to block the target enzyme's activity.

### Mechanism of TMC207/target interaction

Insight in the mode of binding of antibacterial drugs to their target proteins is an important step in understanding the mechanism of drug action. Moreover, new drug derivates may be designed based on knowledge of drug/target interaction [33]. Previously, docking studies based on free energy minimization predicted a binding niche for TMC207 in mycobacterial ATP synthase [27], [33]. This site is mainly made up by subunit c, supplemented with residues from subunit a. More specifically, TMC207 in an extended conformation [34] has been proposed to interact via its protonated basic amino group with the carboxyl group of glutamate61 in subunit c [27], [33].

Our results show that TMC207 binds to a distinct drug-binding site within ATP synthase with electrostatic interactions playing an important role in drug binding. Most likely, the protonated form of TMC207 is the active molecule. These results are consistent with the model proposed by de Jonge et al., as the predicted interaction of the protonated amino group of TMC207 with Glu61 predominantly is electrostatic in character and contributes significantly to efficient drug binding. These electrostatic interactions are expected to be accompanied by hydrophobic and stacking interactions between aromatic rings of TMC207 and aromatic side chains in subunit c [33], which may explain why in our experiments even at high salt concentrations still significant binding was observed. The observed lack of competition between TMC207 and protons for a common binding site suggests that protonated TMC207 may interfere with conformational changes in ATP synthase, e.g. block the rotary motion of subunit c. This result is consistent with the hypothesis that TMC207 prevents rotation of subunit c by mimicking the function of arginine186 in subunit a [27], a conserved basic residue in the proton transfer chain [35].

Taken together, our results are consistent with previous predictions based on docking studies. TMC207, bound in a defined niche at the interface of subunits c and subunit a, may interfere with proton transfer and subsequently block conformational changes associated with ATP synthase activity.

### TMC207 can be active in a broad range of physiological microenvironments

Mycobacteria can persist in a mammalian host in “low energy” environments due to exceptional metabolic flexibility [36], e.g. in poorly aerated parts of the lung, within encapsulated lesions or within the endosome system of host macrophages [37]. Bacteria in these microenvironments are notoriously difficult to kill with antibacterials, such as isoniazid or ethionamide [38], [39]. Previously, it was demonstrated that mycobacteria cultivated *in vitro* in low oxygen tension model systems were efficiently killed by TMC207 [14], [15]. However, in addition to low oxygen tension, mycobacterial microenvironments can display nutrient limitation, which may allow for only a low proton motive force across the cytoplasmatic membrane [40]. Moreover, mammalian granuloma can be acidic due to active inflammation, with pH values as low as 5.0 [9]. The high affinity of TMC207 for its target at both low proton motive force and low pH values may contribute to the drug's ability to render infected tissue culture-negative in mice faster than current first- and second-line antibiotics [10]–[12].

### Conclusion

Our results show that TMC207 binds to a distinct drug-binding site in its target and we provide experimental support for a binding model previously proposed based on docking studies [27]. The drug most likely interferes with proton transfer and blocks conformational changes associated with proton flow.

TMC207 efficiently interacts with its target independent of environmental conditions such as the local pH and the proton motive force. These properties, combined with the essentiality of the target, may explain how TMC207 can act as a highly potent antibacterial drug.
